# Multi-phasic life-threatening anaphylaxis refractory to epinephrine managed by extracorporeal membrane oxygenation (ECMO): A case report

**DOI:** 10.3389/falgy.2022.934436

**Published:** 2022-07-29

**Authors:** Juergen Grafeneder, Florian Ettl, Alexandra-Maria Warenits, Nina Buchtele, Elisabeth Lobmeyr, Thomas Staudinger, Michael Schwameis, Wolfgang R. Sperr, Georg Gelbenegger, Christian Schoergenhofer, Bernd Jilma

**Affiliations:** ^1^Department of Emergency Medicine, Medical University of Vienna, Vienna, Austria; ^2^Intensive Care Unit 13i2, Division of Hematology and Hemostaseology, Department of Internal Medicine I, Medical University of Vienna, Vienna, Austria; ^3^Department of Clinical Pharmacology, Medical University of Vienna, Vienna, Austria

**Keywords:** mastocytosis, MCAS, anaphylactic shock, bronchospasm, respiratory failure, epinephrine resistance, extracorporeal membrane oxygenation (ECMO)

## Abstract

We present a case of a 52-year-old patient suffering from multi-phasic life-threatening anaphylaxis refractory to epinephrine treatment. Extracorporeal membrane oxygenation (ECMO) therapy was initiated as the *ultima ratio* to stabilize the patient hemodynamically during episodic severe bronchospasm. ECMO treatment was successfully weaned after 4 days. Mastocytosis was diagnosed as the underlying condition. Although epinephrine is recommended as a first-line treatment for anaphylaxis, this impressive case provides clear evidence of its limited therapeutic success and emphasizes the need for causal therapies.

## Introduction

Mastocytosis is an orphan disease characterized by an increase of neoplastic mast cells (1). Most patients present with non-advanced mastocytosis and have a normal or near-normal life expectancy. The KIT D816V gene activating mutation is considered central to the pathogenesis and is present in the vast majority (>80%) of systemic mastocytosis (2). This gene regulates the proliferation and differentiation of mast cells. Mediator-related symptoms may include anaphylaxis, bone pain, gastrointestinal problems, fatigue, and osteoporosis. Mast cell activation leads to degranulation of mast cells (3) and subsequent tryptase and histamine release.

## Context

### Initial presentation

Pre-Hospital: A 52-year-old patient without known allergies developed sudden respiratory distress and made an emergency call (12:35 pm). When the paramedics arrived, she was hypoxic and hypotensive (oxygen saturation (SpO2): 87%, respiratory rate: 20/min, blood pressure: 90/50 mmHg, heart rate: 120/min). Oxygen insufflation (10 L/min) was initiated, and she received intravenous phenylephrine, inhaled salbutamol, and ipratropium bromide. The patient's medical history included arterial hypertension (treated with carvedilol 25 mg in the evening), recurring panic attacks (treated with alprazolam 0.5 mg at night), and a subtotal thyroidectomy 30 years ago (100 μg levothyroxine in the morning for substitution). There was no recent change in medication or history of hemodynamic instability. There were no known triggers for this event.

### Clinical course

**Figure 1:**
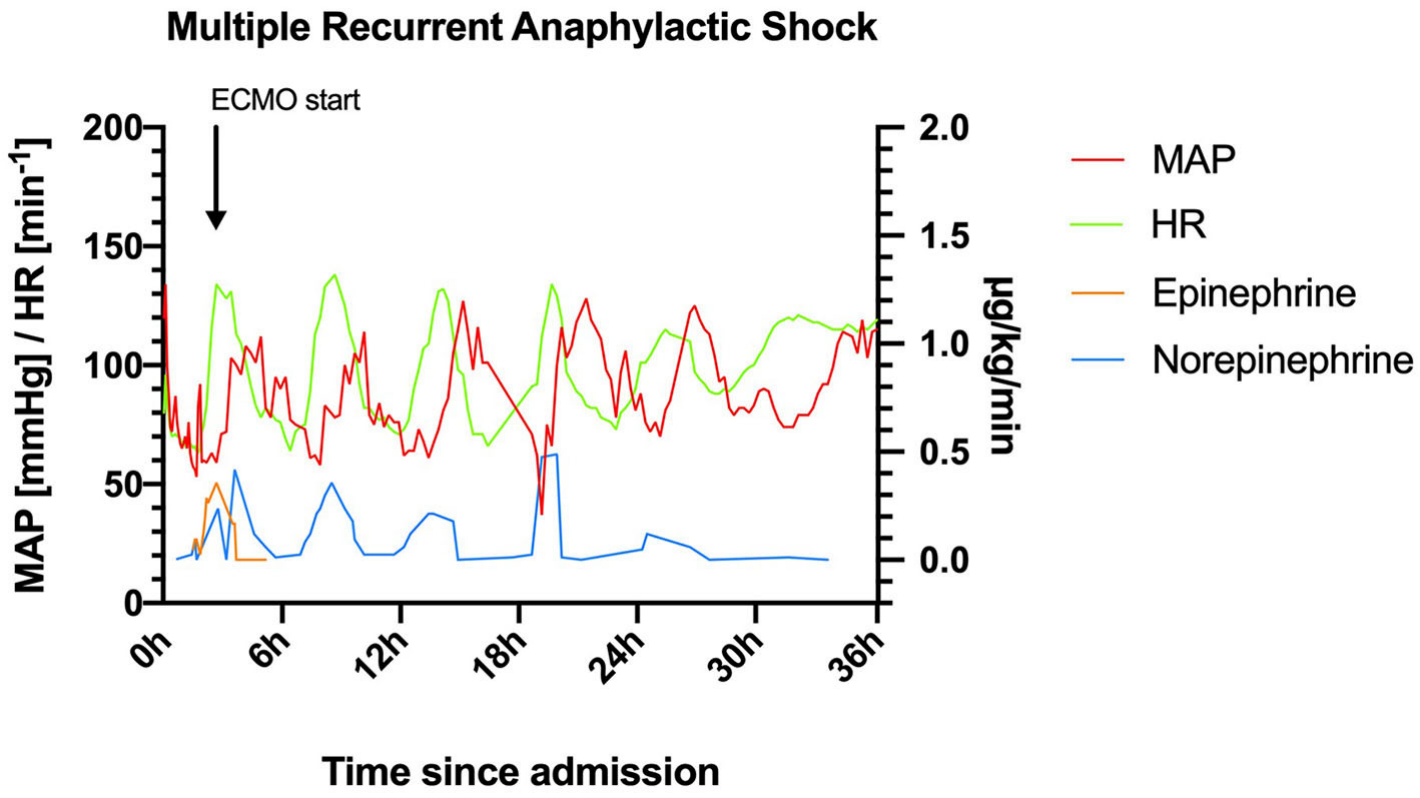
Heart rate (HR) and mean arterial pressure (MAP) within the first 36 h after admission to the intensive care unit. ECMO, extracorporeal membrane oxygenation.

*Day 1:* On arrival at the emergency department (at 1:23 pm) the patient was hemodynamically unstable and oxygen saturation was deteriorating (SpO2 83%) despite oxygen insufflation (15 L/min). Increasing doses of norepinephrine and maximum non-invasive oxygen support failed to stabilize the patient. Following endotracheal intubation and initiation of continuous epinephrine infusion (maximum dose: 0.48 μg/kg/min) instead of norepinephrine, the patient could be stabilized. Contrast-enhanced computed tomography ruled out pulmonary embolism and aortic dissection. Immediately following the scan, the patient developed flush/urticaria, red conjunctivas, and respiratory instability, needing increased respiratory support. Prednisolone 250 mg and the histamine H1 receptor blocker diphenhydramine 60 mg were administered for a suspected anaphylactic reaction. Subsequently, the patient stabilized, and the dose of continuous epinephrine infusion could be substantially reduced (0.01 μg/kg/min). The patient was normotensive (131/79 mmHg) albeit tachycardic (131 min^−1^), and acidotic (pH: 7.23) despite mechanical ventilation (FiO2: 0.7, PEEP: 6 mbar, pressure support: 13 mbar, respiratory rate: 18 min^−1^ under analgo-sedation using propofol and fentanyl) at 6:00 pm. Then, 30 min later, she developed another bout of anaphylaxis (Figure 1) and rapidly deteriorated with a fall in blood pressure (>30%) to 70/47 mmHg. Despite an increase in the continuous epinephrine infusion to 0.357 μg/kg/min, the mean arterial blood pressure did not increase to >60 mmHg during the next hour. Only with the addition of norepinephrine (0.238 μg/kg/min), the blood pressure stabilized at >70 mmHg at around 8:15 pm. However, severe bronchospasm rapidly aggravated the respiratory compromise. Treatment with inhaled fenoterol, ipratropium bromide, and epinephrine as well as intravenously and subcutaneously administered terbutaline sulfate resulted in a brief period of stabilization. Around 9:00 pm another bout of anaphylaxis occurred with flushing, severe bronchospasm, and hemodynamic instability. Stabilization was not achieved despite continuous epinephrine (0.5 μg/kg/min) support, intensified analgo-sedation (addition of ketamine), and muscle relaxation. The systolic blood pressure dropped to 68 mmHg, while the SpO2 stayed above 93%. A venous-arterial (femoral-femoral) extracorporeal membrane oxygenation (ECMO) had to be initiated as an *ultima ratio* for sufficient hemodynamic support (blood flow 2.7 l/min, FiO2 1). Thereafter the patient received another dose of 250 mg methylprednisolone (10:00 pm) and 120 mg diphenhydramine (10:00 pm and 2:00 am). Ketamine was terminated due to lack of effect, and sedation was switched to midazolam alone to test for soy allergy.

**Figure 2:**
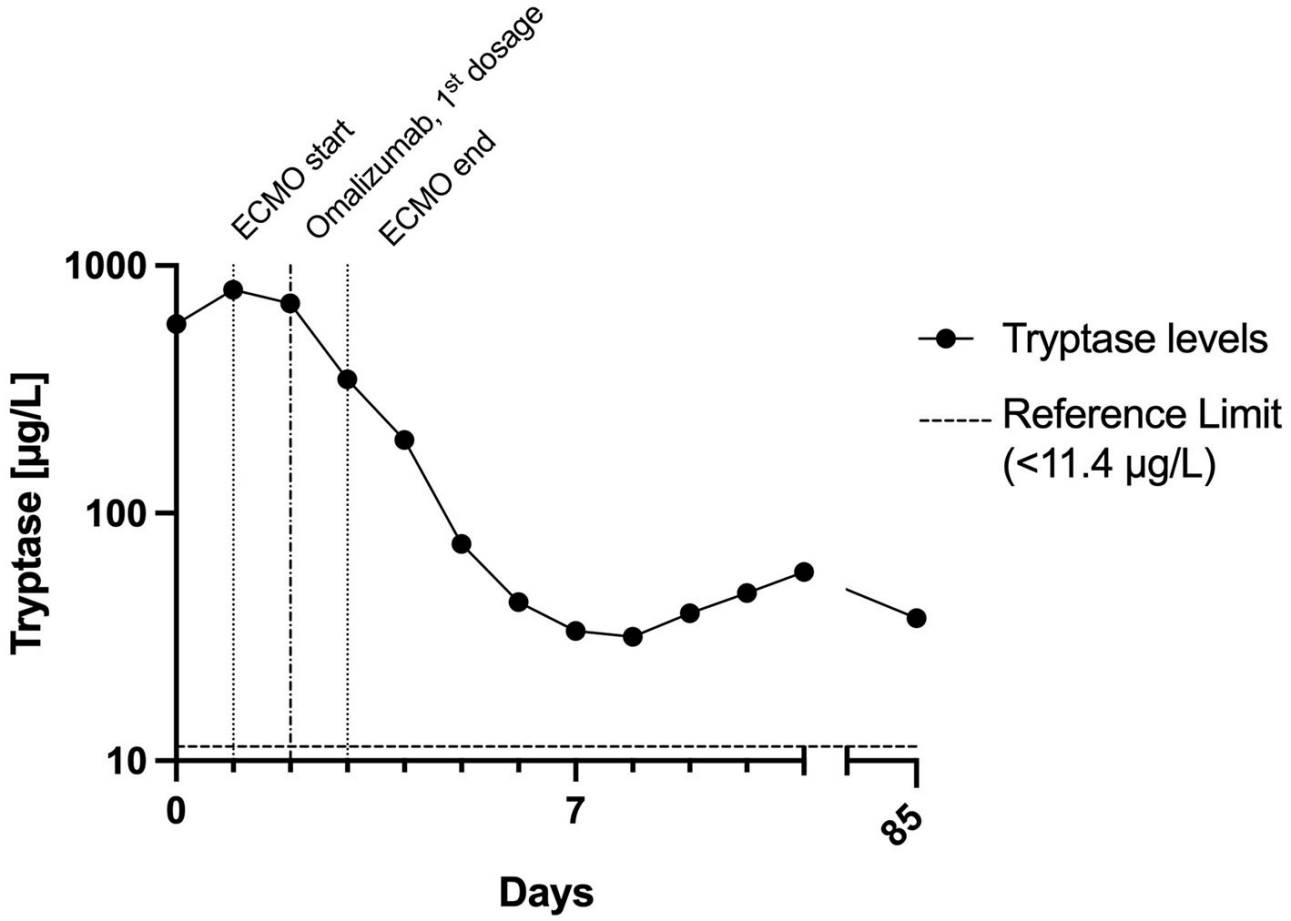
Time course of tryptase levels within the first days and follow-up. The half-life of tryptase between days 2 and 5 was about 32 h. This is in stark contrast to the normal tryptase half-life of 2 h. This indicates ongoing mast cell activation for a couple of days after the initial peak. ECMO, extracorporeal membrane oxygenation.

*Day 2:* The patient suffered several further severe bouts of anaphylaxis, during which the hemodynamic situation was barely manageable despite ECMO (blood flow 3 l/min flow, FiO2 1.0), and ventilation was virtually impossible. Catecholamine and respiratory support were increased substantially during these episodes, and inhalative epinephrine and additional fluid substitution were given. After roughly 24 hours, sedation was switched back to propofol. Severe mast cell activation was suspected because tryptase levels were increased >50-fold the upper normal limit, and omalizumab 300 mg was injected subcutaneously (Figure 2). Later this day the patient suffered another episode of bronchospasm, which resolved after epinephrine inhalation, and systolic blood pressure stabilized at >90 mmHg about 60 h after the onset of anaphylaxis.

*Day 3:* The patient remained stable, catecholamines were tapered (reduced), and respiratory support was decreased.

*Day 4–9:* The ECMO was successfully explanted on day 4 when tachycardia disappeared. The patient was successfully extubated on day 5. The molecular workup of the peripheral blood and the bone marrow revealed the KIT D816V mutation. The bone marrow biopsy (not the smear) showed 40% infiltration with spindle mast cells with the abnormal expression of CD2 and CD25, diagnosing indolent systemic mastocytosis. Prophylactic treatment with antihistamines was instigated. The patient was transferred to a normal ward as no further events occurred.

Normal ward: The patient remained stable for another 13 days on the normal ward and was discharged in good health.

## Discussion

This patient with mastocytosis developed multiphasic severe anaphylactic shocks; including the pre-hospital phase, 6 episodes of anaphylaxis occurred within 30 h with a remarkable rhythm (Figure 1).

The ECMO support was vital due to repeated life-threatening hypoxia caused by bronchospasms and severe circulatory instability. Although histamine plays a key role in bronchospasm, antihistamines remain controversial in anaphylaxis. The likely reason for their ineffectiveness is that they are easily overwhelmed by excessive histamine concentrations (4) >5-fold the upper normal limit (<1 ng/mL) (5), whereas in severe anaphylaxis histamine concentrations of >100 ng/mL have been reported (6). A Cochrane systematic review found no evidence of antihistamine efficacy in anaphylaxis (7). In this case the patient's condition worsened despite the repeated infusion of antihistamines and glucocorticoids.

Epinephrine is recommended as a first-line treatment for anaphylaxis (8), although there is no supporting evidence from randomized controlled trials. To our knowledge, there is no study investigating the role of epinephrine in the treatment of mast cell activation syndrome. It appears that up-titration of epinephrine to 0.357 μg/kg/min over 50 min neither stabilized the mean arterial pressure above 60 mmHg nor prevented the subsequent severe bronchospasm. An ECMO implantation was required. Thus, even intravenous epinephrine may not provide the desired benefit in anaphylactic shock or respiratory failure.

Omalizumab binds to free IgE, thus reducing mast cell activation. The decrease in symptoms and tryptase levels after omalizumab should not be interpreted as a cause-effect relationship. Omalizumab was administered after the first 5 episodes of tachycardia, and the patient remained tachycardiac for another 24 h. In patients with mastocytosis who respond to treatment, it takes a median of 1 month for symptoms to improve (9).

In conclusion, anaphylaxis in systemic mastocytosis can lead to a life-threatening event that is unresponsive to conventional therapy including epinephrine. Supportive therapy with ECMO for hemodynamic shock and respiratory failure may be lifesaving, and more effective therapies are needed (10).

## Data Availability Statement

The original contributions presented in the study are included in the article/Supplementary material, further inquiries can be directed to the corresponding author/s.

## Ethics Statement

Ethical review and approval was not required for the study on human participants in accordance with the local legislation and institutional requirements. Written informed consent was obtained from the patient for the publication of this case report.

## Author contributions

TS, MS, WS, CS, and BJ contributed to conception and design of the case report. JG, FE, and A-MW wrote the first draft of the manuscript. NB, EL, and GG wrote sections of the manuscript. All authors contributed to manuscript revision, read, and approved the submitted version.

## Conflict of interest

The Medical University of Vienna has been granted a patent for recombinant human diamine oxidase, on which BJ is listed among the inventors. The remaining authors declare that the research was conducted in the absence of any commercial or financial relationships that could be construed as a potential conflict of interest.

## Publisher's note

All claims expressed in this article are solely those of the authors and do not necessarily represent those of their affiliated organizations, or those of the publisher, the editors and the reviewers. Any product that may be evaluated in this article, or claim that may be made by its manufacturer, is not guaranteed or endorsed by the publisher.
