# Supplementary material for: Multi-phasic life-threatening anaphylaxis refractory to epinephrine managed by extracorporeal membrane oxygenation (ECMO): A case report
Source: Front Allergy. 2022 Jul 29;3:934436. doi: 10.3389/falgy.2022.934436 (PMC9372331; doi:10.3389/falgy.2022.934436)
Supplement: Supplementary file 1 [file Image_1.pdf]

Supplemental Figure 1. Vital Parameters of the patient within the first 72h hours after admission to the ICU. A) Heart rate and MAP; B) Heart rate and SpO2; C) MAP and SpO2; D) ECMO FiO2 and SpO2.

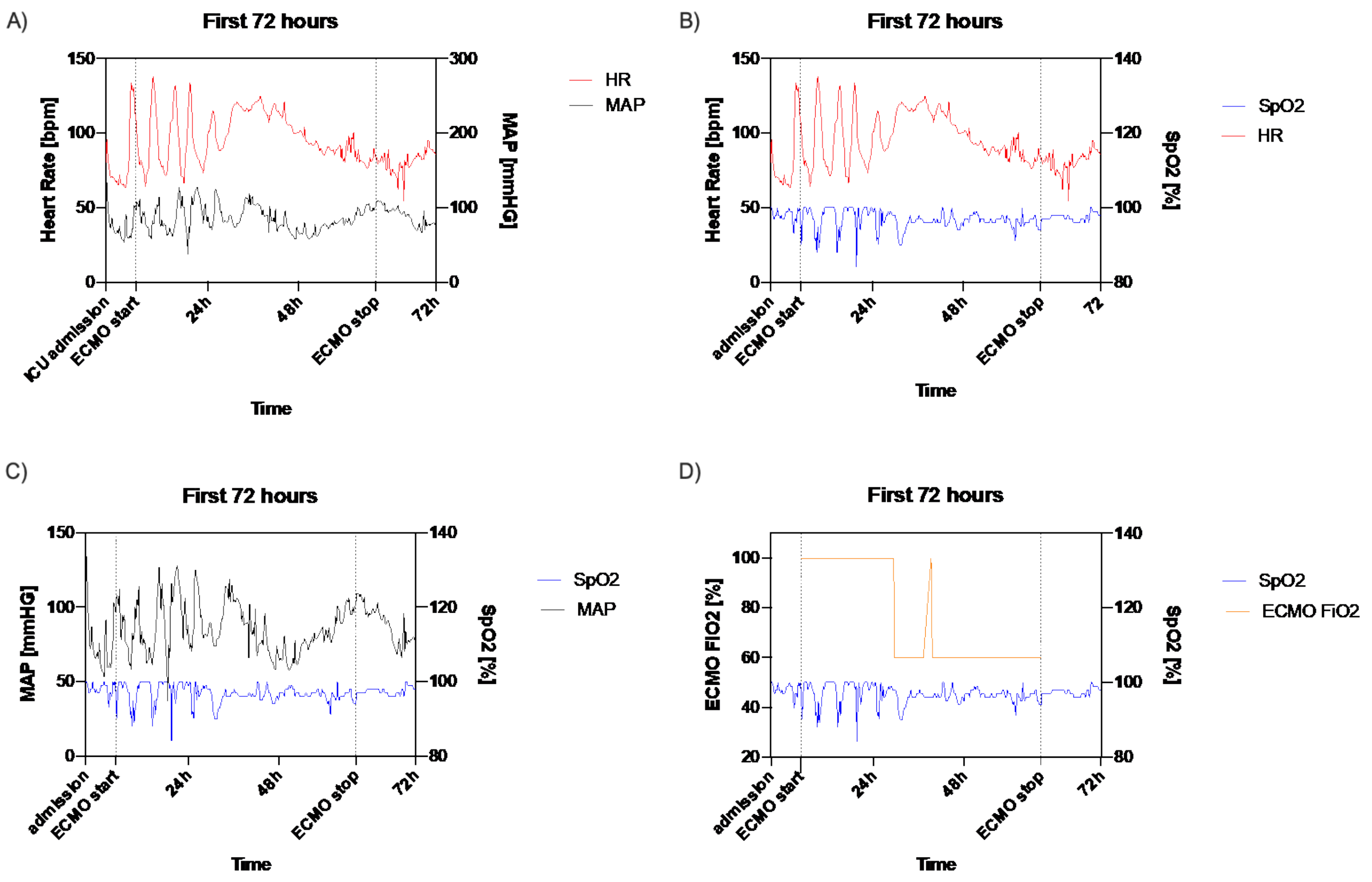

Bpm … beats per minute; HR … Heart Rate; MAP … mean arterial Pressure; mmHg … millimeters of mercury: ECMO … extracorporeal membrane oxygenation; ICU … intensive care unit; SpO2 … Saturation of peripheral Oxygen; FiO2 ... Fraction of Inspired Oxygen
